# 1-Propyl-1*H*-2,1-benzothia­zin-4(3*H*)-one 2,2-dioxide

**DOI:** 10.1107/S160053681004078X

**Published:** 2010-10-20

**Authors:** Islam Ullah Khan, Muhammad Shafiq, Muhammad Nadeem Arshad

**Affiliations:** a Materials Chemistry laboratory, Department of Chemistry, GC University, Lahore 54000, Pakistan

## Abstract

In the title compound, C_11_H_13_NO_3_S, a benzothia­zine derivative, the heterocycle adopts a sofa conformation. In the crystal, weak C—H⋯O hydrogen bonds connect the mol­ecules into a three-dimensional network.

## Related literature

For the synthesis of the title compound, see: Volovenko *et al.* (2007 ▶). For a related structure, see: Shafiq *et al.* (2009 ▶).
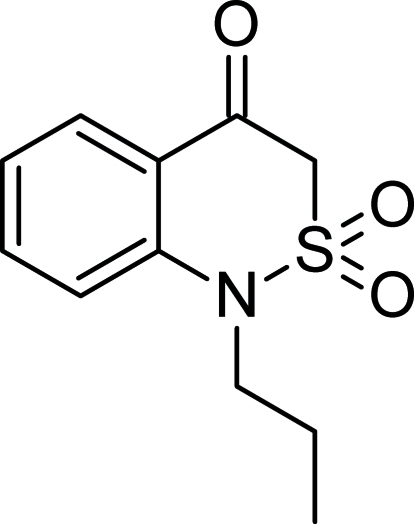


## Experimental

### 

#### Crystal data

C_11_H_13_NO_3_S
*M* *_r_* = 239.28
Triclinic, 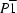

*a* = 7.9448 (2) Å
*b* = 8.0701 (3) Å
*c* = 9.6267 (2) Å
α = 87.468 (2)°
β = 84.097 (2)°
γ = 64.453 (1)°
*V* = 553.92 (2) Å^3^
*Z* = 2
Mo *K*α radiation
μ = 0.28 mm^−1^
*T* = 296 K
0.28 × 0.21 × 0.12 mm

#### Data collection

Bruker Kappa APEXII CCD diffractometer
Absorption correction: multi-scan (*SADABS*; Bruker, 2007 ▶) *T*_min_ = 0.925, *T*_max_ = 0.967
12058 measured reflections
2765 independent reflections
2229 reflections with *I* > 2σ(*I*)
*R*_int_ = 0.027

#### Refinement

*R*[*F* ^2^ > 2σ(*F* ^2^)] = 0.038
*wR*(*F* ^2^) = 0.111
*S* = 1.07
2765 reflections
146 parameters
H-atom parameters constrained
Δρ_max_ = 0.30 e Å^−3^
Δρ_min_ = −0.38 e Å^−3^

### 

Data collection: *APEX2* (Bruker, 2007 ▶); cell refinement: *SAINT* (Bruker, 2007 ▶); data reduction: *SAINT*; program(s) used to solve structure: *SHELXS97* (Sheldrick, 2008 ▶); program(s) used to refine structure: *SHELXL97* (Sheldrick, 2008 ▶); molecular graphics: *ORTEP-3 for Windows* (Farrugia, 1997 ▶) and *PLATON* (Spek, 2009 ▶); software used to prepare material for publication: *WinGX* (Farrugia, 1999 ▶) and *PLATON*.

## Supplementary Material

Crystal structure: contains datablocks I, global. DOI: 10.1107/S160053681004078X/bt5369sup1.cif

Structure factors: contains datablocks I. DOI: 10.1107/S160053681004078X/bt5369Isup2.hkl

Additional supplementary materials:  crystallographic information; 3D view; checkCIF report

## Figures and Tables

**Table 1:** Hydrogen-bond geometry (Å, °)

*D*—H⋯*A*	*D*—H	H⋯*A*	*D*⋯*A*	*D*—H⋯*A*
C11—H11*B*⋯O2^i^	0.96	2.57	3.346 (2)	138
C8—H8*A*⋯O3^ii^	0.97	2.55	3.453 (2)	155
C2—H2⋯O1^iii^	0.93	2.55	3.4665 (19)	170

Symmetry codes: (i) 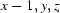; (ii) 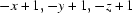; (iii) 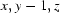.

**Fig. 1.**
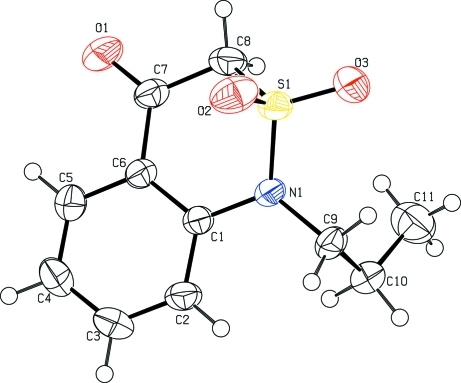
Molecular structure of the title compound with displacement ellipsoids drawn at the 50% probability level.

**Fig. 2.**
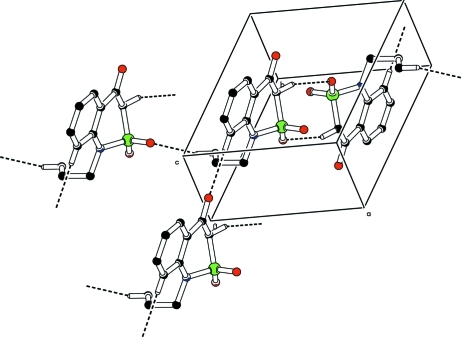
Unit cell packing diagram showing the hydrogen bonding with dashed lines.

## supplementary crystallographic information

### Comment

Here we report the crystal structure of title compound in countinuation to the previously published 3,3-dichloro-1-ethyl-1*H*-2,1-benzothiazin-4(3*H*)-one 2,2-dioxide derivative (Shafiq *et al.*, 2009). The difference between the two compounds is a propyl which differ just only in substitution at N and at the methylene C atom in the benzothiazine ring. The heterocycle adopts a sofa conformation. Weak C—H···O type hydrogen bonds connect the molecules to a  three dimensional network.

### Experimental

The title compound was prepared following the available literature procedure (Volovenko *et al.*, 2007).

### Refinement

All the C—H H-atoms were positioned with idealized geometry with C—H = 0.93 Å for aromatic C—H = 0.97 Å for methylene C—H = 0.96 Å for methyl and were refined using a riding model with *U*_iso_(H) = 1.2 *U*_eq_(C) for aromatic, with *U*_iso_(H) = 1.2 *U*_eq_(C) for methylene, with *U*_iso_(H) = 1.5 *U*_eq_(C) for methyl.

### Crystal data

**Table:**

C_11_H_13_NO_3_S	*Z* = 2
*M**_r_* = 239.28	*F*(000) = 252
Triclinic, *P*1	*D*_x_ = 1.435 Mg m^−^^3^
Hall symbol: -P 1	Mo *K*α radiation, λ = 0.71073 Å
*a* = 7.9448 (2) Å	Cell parameters from 5097 reflections
*b* = 8.0701 (3) Å	θ = 2.2–21.8°
*c* = 9.6267 (2) Å	µ = 0.28 mm^−^^1^
α = 87.468 (2)°	*T* = 296 K
β = 84.097 (2)°	Needle, light brown
γ = 64.453 (1)°	0.28 × 0.21 × 0.12 mm
*V* = 553.92 (2) Å^3^	

### Data collection

**Table:**

Bruker Kappa APEXII CCD diffractometer	2765 independent reflections
Radiation source: fine-focus sealed tube	2229 reflections with *I* > 2σ(*I*)
graphite	*R*_int_ = 0.027
φ and ω scans	θ_max_ = 28.3°, θ_min_ = 2.1°
Absorption correction: multi-scan (*SADABS*; Bruker, 2007)	*h* = −10→10
*T*_min_ = 0.925, *T*_max_ = 0.967	*k* = −10→10
12058 measured reflections	*l* = −12→12

### Refinement

**Table:**

Refinement on *F*^2^	Primary atom site location: structure-invariant direct methods
Least-squares matrix: full	Secondary atom site location: difference Fourier map
*R*[*F*^2^ > 2σ(*F*^2^)] = 0.038	Hydrogen site location: inferred from neighbouring sites
*wR*(*F*^2^) = 0.111	H-atom parameters constrained
*S* = 1.07	*w* = 1/[σ^2^(*F*_o_^2^) + (0.0604*P*)^2^ + 0.0882*P*] where *P* = (*F*_o_^2^ + 2*F*_c_^2^)/3
2765 reflections	(Δ/σ)_max_ < 0.001
146 parameters	Δρ_max_ = 0.30 e Å^−^^3^
0 restraints	Δρ_min_ = −0.38 e Å^−^^3^

### Special details

**Table:**

Geometry. All s.u.'s (except the s.u. in the dihedral angle between two l.s. planes) are estimated using the full covariance matrix. The cell s.u.'s are taken into account individually in the estimation of s.u.'s in distances, angles and torsion angles; correlations between s.u.'s in cell parameters are only used when they are defined by crystal symmetry. An approximate (isotropic) treatment of cell s.u.'s is used for estimating s.u.'s involving l.s. planes.
Refinement. Refinement of *F*^2^ against ALL reflections. The weighted *R*-factor *wR* and goodness of fit *S* are based on *F*^2^, conventional *R*-factors *R* are based on *F*, with *F* set to zero for negative *F*^2^. The threshold expression of *F*^2^ > 2σ(*F*^2^) is used only for calculating *R*-factors(gt) *etc*. and is not relevant to the choice of reflections for refinement. *R*-factors based on *F*^2^ are statistically about twice as large as those based on *F*, and *R*- factors based on ALL data will be even larger.

### Fractional atomic coordinates and isotropic or equivalent isotropic displacement parameters (Å^2^)

**Table:**

	*x*	*y*	*z*	*U*_iso_*/*U*_eq_	
S1	0.44648 (6)	0.31465 (5)	0.66978 (4)	0.04181 (14)	
O1	0.2757 (2)	0.71661 (17)	0.91660 (14)	0.0633 (4)	
O2	0.62535 (17)	0.25460 (17)	0.71992 (15)	0.0584 (3)	
O3	0.4391 (2)	0.2879 (2)	0.52525 (12)	0.0680 (4)	
N1	0.31827 (18)	0.22605 (17)	0.76115 (12)	0.0380 (3)	
C1	0.28023 (19)	0.26527 (18)	0.90617 (14)	0.0313 (3)	
C2	0.2496 (2)	0.1412 (2)	0.99954 (16)	0.0408 (3)	
H2	0.2519	0.0338	0.9661	0.049*	
C3	0.2161 (2)	0.1770 (2)	1.14058 (16)	0.0473 (4)	
H3	0.1951	0.0935	1.2012	0.057*	
C4	0.2129 (2)	0.3344 (2)	1.19393 (17)	0.0494 (4)	
H4	0.1947	0.3549	1.2898	0.059*	
C5	0.2372 (2)	0.4602 (2)	1.10331 (16)	0.0421 (3)	
H5	0.2322	0.5679	1.1384	0.051*	
C6	0.26911 (19)	0.42939 (18)	0.95988 (14)	0.0323 (3)	
C7	0.2874 (2)	0.57493 (19)	0.87064 (16)	0.0376 (3)	
C8	0.3163 (3)	0.5469 (2)	0.71447 (16)	0.0447 (4)	
H8A	0.3812	0.6171	0.6723	0.054*	
H8B	0.1953	0.5924	0.6773	0.054*	
C9	0.3171 (2)	0.0585 (2)	0.70536 (16)	0.0398 (3)	
H9A	0.3717	−0.0425	0.7696	0.048*	
H9B	0.3936	0.0264	0.6168	0.048*	
C10	0.1214 (2)	0.0854 (2)	0.68464 (17)	0.0461 (4)	
H10A	0.0411	0.1345	0.7699	0.055*	
H10B	0.1236	−0.0331	0.6673	0.055*	
C11	0.0384 (3)	0.2133 (3)	0.5651 (2)	0.0662 (5)	
H11A	0.0339	0.3315	0.5821	0.099*	
H11B	−0.0860	0.2258	0.5575	0.099*	
H11C	0.1149	0.1637	0.4797	0.099*	

### Atomic displacement parameters (Å^2^)

**Table:**

	*U*^11^	*U*^22^	*U*^33^	*U*^12^	*U*^13^	*U*^23^
S1	0.0535 (3)	0.0410 (2)	0.0377 (2)	−0.02944 (19)	0.01063 (16)	−0.00505 (15)
O1	0.0991 (11)	0.0410 (7)	0.0630 (8)	−0.0439 (7)	0.0020 (7)	−0.0069 (6)
O2	0.0442 (7)	0.0516 (7)	0.0801 (9)	−0.0240 (6)	0.0097 (6)	−0.0051 (6)
O3	0.1086 (11)	0.0773 (9)	0.0367 (7)	−0.0615 (9)	0.0177 (7)	−0.0119 (6)
N1	0.0526 (7)	0.0369 (6)	0.0339 (6)	−0.0295 (6)	0.0055 (5)	−0.0063 (5)
C1	0.0338 (7)	0.0295 (7)	0.0321 (7)	−0.0157 (5)	−0.0007 (5)	−0.0004 (5)
C2	0.0499 (9)	0.0319 (7)	0.0438 (8)	−0.0219 (7)	0.0001 (6)	0.0035 (6)
C3	0.0532 (10)	0.0467 (9)	0.0407 (9)	−0.0222 (8)	−0.0008 (7)	0.0127 (7)
C4	0.0580 (10)	0.0567 (10)	0.0307 (7)	−0.0227 (8)	−0.0012 (7)	0.0008 (7)
C5	0.0479 (9)	0.0413 (8)	0.0382 (8)	−0.0201 (7)	−0.0012 (6)	−0.0070 (6)
C6	0.0338 (7)	0.0294 (7)	0.0353 (7)	−0.0151 (5)	−0.0021 (5)	−0.0008 (5)
C7	0.0419 (8)	0.0297 (7)	0.0438 (8)	−0.0184 (6)	−0.0010 (6)	−0.0004 (6)
C8	0.0595 (10)	0.0351 (8)	0.0426 (8)	−0.0249 (7)	0.0003 (7)	0.0064 (6)
C9	0.0488 (9)	0.0321 (7)	0.0419 (8)	−0.0211 (6)	0.0016 (6)	−0.0089 (6)
C10	0.0573 (10)	0.0497 (9)	0.0436 (8)	−0.0347 (8)	−0.0039 (7)	−0.0002 (7)
C11	0.0646 (12)	0.0862 (15)	0.0562 (11)	−0.0401 (11)	−0.0128 (9)	0.0161 (10)

### Geometric parameters (Å, °)

**Table:**

S1—O2	1.4191 (14)	C5—C6	1.3915 (19)
S1—O3	1.4282 (13)	C5—H5	0.9300
S1—N1	1.6464 (12)	C6—C7	1.473 (2)
S1—C8	1.7535 (16)	C7—C8	1.509 (2)
O1—C7	1.2069 (18)	C8—H8A	0.9700
N1—C1	1.4178 (17)	C8—H8B	0.9700
N1—C9	1.4808 (17)	C9—C10	1.508 (2)
C1—C2	1.3982 (19)	C9—H9A	0.9700
C1—C6	1.4070 (18)	C9—H9B	0.9700
C2—C3	1.375 (2)	C10—C11	1.513 (3)
C2—H2	0.9300	C10—H10A	0.9700
C3—C4	1.380 (2)	C10—H10B	0.9700
C3—H3	0.9300	C11—H11A	0.9600
C4—C5	1.373 (2)	C11—H11B	0.9600
C4—H4	0.9300	C11—H11C	0.9600
			
O2—S1—O3	117.98 (9)	O1—C7—C6	122.99 (14)
O2—S1—N1	111.54 (7)	O1—C7—C8	118.84 (13)
O3—S1—N1	107.86 (7)	C6—C7—C8	118.14 (12)
O2—S1—C8	107.87 (8)	C7—C8—S1	111.75 (10)
O3—S1—C8	110.27 (9)	C7—C8—H8A	109.3
N1—S1—C8	99.80 (7)	S1—C8—H8A	109.3
C1—N1—C9	120.81 (11)	C7—C8—H8B	109.3
C1—N1—S1	116.96 (9)	S1—C8—H8B	109.3
C9—N1—S1	117.37 (10)	H8A—C8—H8B	107.9
C2—C1—C6	118.28 (13)	N1—C9—C10	111.75 (13)
C2—C1—N1	120.18 (12)	N1—C9—H9A	109.3
C6—C1—N1	121.53 (12)	C10—C9—H9A	109.3
C3—C2—C1	120.42 (14)	N1—C9—H9B	109.3
C3—C2—H2	119.8	C10—C9—H9B	109.3
C1—C2—H2	119.8	H9A—C9—H9B	107.9
C2—C3—C4	121.32 (14)	C9—C10—C11	113.28 (15)
C2—C3—H3	119.3	C9—C10—H10A	108.9
C4—C3—H3	119.3	C11—C10—H10A	108.9
C5—C4—C3	118.96 (14)	C9—C10—H10B	108.9
C5—C4—H4	120.5	C11—C10—H10B	108.9
C3—C4—H4	120.5	H10A—C10—H10B	107.7
C4—C5—C6	121.20 (14)	C10—C11—H11A	109.5
C4—C5—H5	119.4	C10—C11—H11B	109.5
C6—C5—H5	119.4	H11A—C11—H11B	109.5
C5—C6—C1	119.74 (13)	C10—C11—H11C	109.5
C5—C6—C7	117.26 (12)	H11A—C11—H11C	109.5
C1—C6—C7	122.99 (12)	H11B—C11—H11C	109.5
			
O2—S1—N1—C1	59.41 (12)	C2—C1—C6—C5	3.0 (2)
O3—S1—N1—C1	−169.51 (11)	N1—C1—C6—C5	−178.04 (13)
C8—S1—N1—C1	−54.35 (12)	C2—C1—C6—C7	−176.18 (14)
O2—S1—N1—C9	−96.15 (12)	N1—C1—C6—C7	2.8 (2)
O3—S1—N1—C9	34.93 (14)	C5—C6—C7—O1	−0.1 (2)
C8—S1—N1—C9	150.09 (12)	C1—C6—C7—O1	179.13 (15)
C9—N1—C1—C2	3.0 (2)	C5—C6—C7—C8	−178.19 (13)
S1—N1—C1—C2	−151.64 (12)	C1—C6—C7—C8	1.0 (2)
C9—N1—C1—C6	−175.92 (13)	O1—C7—C8—S1	148.80 (14)
S1—N1—C1—C6	29.41 (17)	C6—C7—C8—S1	−32.99 (17)
C6—C1—C2—C3	−2.2 (2)	O2—S1—C8—C7	−61.58 (13)
N1—C1—C2—C3	178.80 (14)	O3—S1—C8—C7	168.29 (11)
C1—C2—C3—C4	−0.5 (2)	N1—S1—C8—C7	54.98 (12)
C2—C3—C4—C5	2.4 (3)	C1—N1—C9—C10	82.62 (17)
C3—C4—C5—C6	−1.6 (3)	S1—N1—C9—C10	−122.81 (12)
C4—C5—C6—C1	−1.1 (2)	N1—C9—C10—C11	70.53 (19)
C4—C5—C6—C7	178.08 (15)		

### Hydrogen-bond geometry (Å, °)

**Table:**

*D*—H···*A*	*D*—H	H···*A*	*D*···*A*	*D*—H···*A*
C11—H11B···O2^i^	0.96	2.57	3.346 (2)	138
C8—H8A···O3^ii^	0.97	2.55	3.453 (2)	155
C2—H2···O1^iii^	0.93	2.55	3.4665 (19)	170

Symmetry codes: (i) *x*−1, *y*, *z*; (ii) −*x*+1, −*y*+1, −*z*+1; (iii) *x*, *y*−1, *z*.
